# An Overview of Zika Virus and Zika Virus Induced Neuropathies

**DOI:** 10.3390/ijms26010047

**Published:** 2024-12-24

**Authors:** Abdul Wahaab, Bahar E Mustafa, Muddassar Hameed, Hira Batool, Hieu Tran Nguyen Minh, Abdul Tawaab, Anam Shoaib, Jianchao Wei, Jason L. Rasgon

**Affiliations:** 1 Department of Entomology, Pennsylvania State University, University Park, PA 16802, USA; wahaaabwahaaab@gmail.com (A.W.); hmt5396@psu.edu (H.T.N.M.); 2 Department of Biochemistry and Molecular Biology, Pennsylvania State University, University Park, PA 16802, USA; 3 The Center for Infectious Disease Dynamics, Pennsylvania State University, University Park, PA 16802, USA; 4 The Huck Institutes of the Life Sciences, Pennsylvania State University, University Park, PA 16802, USA; 5 School of Veterinary Science, Faculty of Science, The University of Melbourne, Melbourne, VIC 3030, Australia; mustafab@student.unimelb.edu.au; 6 Sub Campus Toba Tek Singh, University of Agriculture, Faisalabad 36050, Pakistan; tawaaab94@gmail.com; 7 Department of Biomedical Sciences and Pathobiology, VA-MD Regional College of Veterinary Medicine, Virginia Polytechnic Institute and State University, Blacksburg, VA 24060, USA; hameed@wustl.edu; 8 Center for Zoonotic and Arthropod-Borne Pathogens, Virginia Polytechnic Institute and State University, Blacksburg, VA 24060, USA; 9 Department of Otolaryngology-Head and Neck Surgery, Department of Pathology and Immunology, Alvin J. Siteman Cancer Center, Washington University School of Medicine, Saint Louis, MO 63110, USA; 10 Chughtai Lab, Head Office, 7-Jail Road, Main Gulberg, Lahore 54000, Pakistan; hirabatoolgmc@gmail.com; 11 School of Behavioral and Brain Sciences, University of Texas at Dallas, Richardson, TX 75080, USA; anamshoaib45@gmail.com; 12 Shanghai Veterinary Research Institute, Chinese Academy of Agricultural Sciences, Shanghai 200241, China; jianchaowei@shvri.ac.cn

**Keywords:** neurotropic flaviviruses, zika virus, neuropathies, transmission, neuropathogenesis, prevention, vaccines, treatment, antivirals, mosquito/vector control, oncolytic, animal and cell models

## Abstract

Flaviviruses pose a major public health concern across the globe. Among them, Zika virus (ZIKV) is an emerging and reemerging arthropod-borne flavivirus that has become a major international public health problem following multiple large outbreaks over the past two decades. The majority of infections caused by ZIKV exhibit mild symptoms. However, the virus has been found to be associated with a variety of congenital neural abnormalities, including microcephaly in children and Guillain–Barre syndrome in adults. The exact prediction of the potential of ZIKV transmission is still enigmatic and underlines the significance of routine detection of the virus in suspected areas. ZIKV transmission from mother to fetus (including fetal abnormalities), viral presence in immune-privileged areas, and sexual transmission demonstrate the challenges in understanding the factors governing viral persistence and pathogenesis. This review illustrates the transmission patterns, epidemiology, control strategies (through vaccines, antivirals, and vectors), oncolytic aspects, molecular insights into neuro-immunopathogenesis, and other neuropathies caused by ZIKV. Additionally, we summarize in vivo and in vitro models that could provide an important platform to study ZIKV pathogenesis and the underlying governing cellular and molecular mechanisms.

## 1. Introduction

Flaviviruses (family *Flaviviridae*) are small (approximately 50 nm in diameter), enveloped, icosahedral viruses that possess a positive sense single-stranded RNA genome of between 9–12 kb [1,2,3,4]. The viral genome consists of a single long ORF (open reading frame) encoding around ten proteins, which are formed by co- and post-translational processing and proteolytically processed into three structural (Cap, prM, and E) and seven non-structural (NS1, NS2A, NS2B, NS3, NS4A, NS4B, and NS5) proteins by a complex combination of host and viral proteases [5,6,7,8]. Currently, there are about 70 members in this genus, the majority of which are transmitted by arthropods [9,10,11,12,13,14,15,16]. The incidence of flaviviral infections has increased dramatically in the last seven decades [17,18]. These infections are associated with significant morbidity and mortality in humans. Factors responsible for the rapid increase in flaviviral infections are changes in the sizes and distributions of the vector arthropods that transmit the pathogens, further aggravated by climate change and a large number of potential vertebrate hosts [19,20,21,22]. Details of various neurotropic flaviviruses and their associated neuropathies are summarized in Table 1.

**Figure 1:**
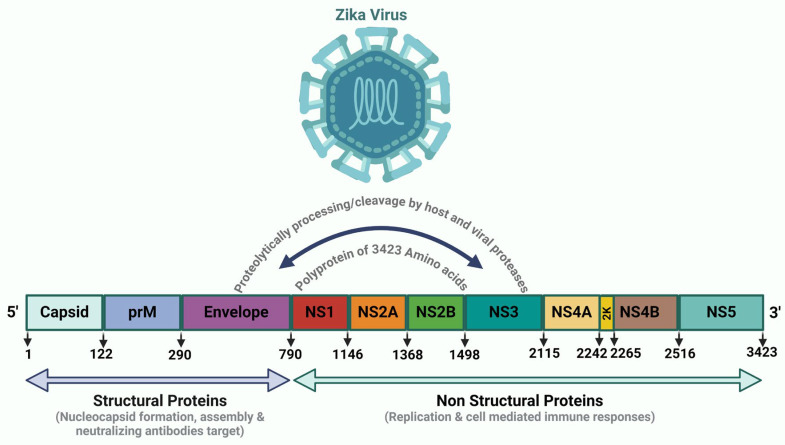
Genome Organization of Zika Virus: The ZIKV genome is composed of 10,794 nucleotides in a single-stranded, positive-sense RNA that encodes a polyprotein of 3423 amino acids and 10 proteins essential for the viral life cycle. ZIKV RNA has two untranslated regions (UTRs) and a single open reading frame (ORF) comprising three structural (Cap, prM, and E) and seven non-structural (NS1, NS2A, NS2B, NS3, NS4A, NS4B, and NS5) proteins [60,61,62].

ZIKV is a significant pathogen that poses an imminent global threat. It was first isolated from rhesus monkeys in Uganda in 1947 [53] (The genome organization of the Zika Virus is sketched in Figure 1). Later, in the 1950s, its detection continued in several other parts of Africa (Egypt, Nigeria) [54]. However, the first clinical infection in humans was not detected until 1964, when it was found that the ZIKV infection in humans is associated with mild fever and maculopapular rash [55]. From 1960 to 2000, ZIKV was detected in several countries in Asia (Malaysia, Indonesia and Pakistan) [54]. The first major ZIKV outbreak occurred in 2007 (in Yap Island), and then in March 2015, it caused a large outbreak in the Americas (particularly Brazil), and the first time was associated with severe disease and congenital illnesses such as infant microcephaly [56,57,58]. In November 2015, Brazil declared a national public health emergency, and in February 2016, the WHO (World Health Organization) declared it a Public Health Emergency of International Concern [59].

**Figure 2:**
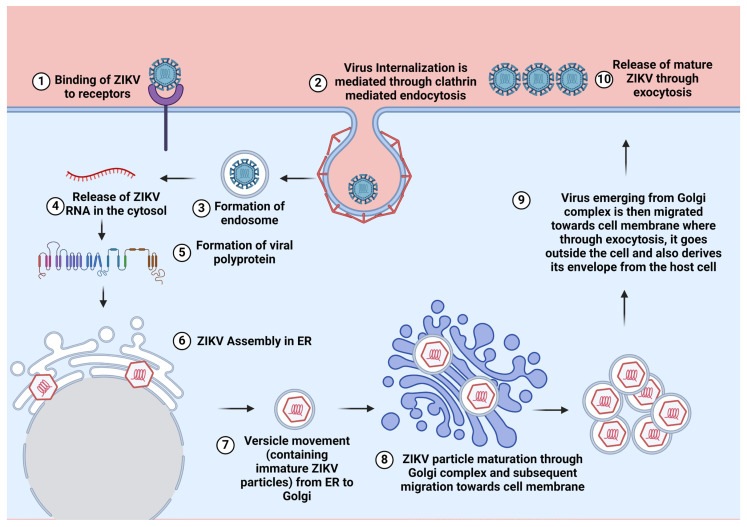
Replication cycle of Zika Virus: The ZIKV replication in the cell is mediated through various steps. (1). The binding of the ZIKV to the host cell is mediated by the interaction of ZIKV E protein and host cell receptors (TIM, TAM, Axl, etc.). (2, 3) Following viral binding to the receptor, the internalization of viral particles inside the cells is mediated through clathrin-coated vesicles, which leads to the formation of endosomes. (4) Inside the endosome, viral E protein rearranges as trimmers that cause the release of ZIKV nucleocapsid in the host cells (5, 6). Viral RNA then produces ZIKV polyprotein, which then enters the ER for the biosynthesis of viral structural and non-structural proteins (NSPs) (7). The immature ZIKV particles then bud off from the ER and migrate towards the Golgi complex. In the Golgi complex, further necessary modifications (glycosylation and others) happen that result in viral maturation. ZIKV then migrates from the Golgi complex to the cell membrane where it leaves the cells through the process of budding (8, 9, 10) [72,73,74,75]. The figure was created at https://BioRender.com.

The ZIKV employs multiple mechanisms to replicate (the ZIKV replication cycle is illustrated in Figure 2) and causes a variety of neurological diseases. It has been proposed that after viral entry through the crossing of the BBB (blood–brain barrier), ZIKV tends to infect the microglia, astrocytes, and endothelial cells [63,64,65]. Damage to the CNS can occur when ZIKV infects the CNS cells, and damage occurs either due to viral replication or because of a host-mediated immune (inflammatory) response [66,67,68]. Moreover, ZIKV infection is also associated with various neurological syndromes, e.g., Guillain–Barré syndrome (GBS), Congenital Zika syndrome (CZS), and others [69,70,71]. Multiple mechanisms are known to cause damage to the CNS, which are also discussed in detail later in this review article. Collectively, in this review, we have summarized the observations on biology, transmission, control strategies (vaccines, antivirals, and vectors), oncolytic aspects, animal and cellular models, neuropathies, and neuropathogenesis associated with ZIKV.

## 2. Transmission of Zika Virus

**Figure 3:**
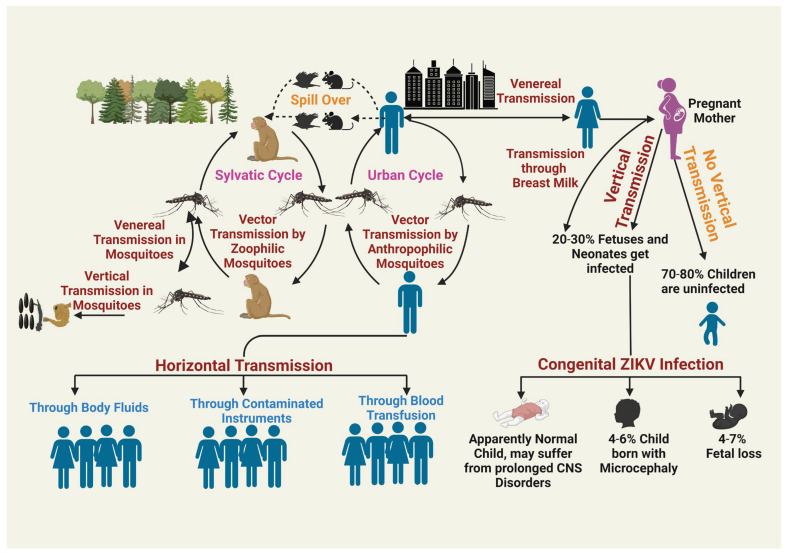
Transmission cycle of Zika Virus: This figure illustrates the transmission of ZIKV. There are two types of ZIKV transmission: Vertical and horizontal transmission. Vertical transmission has been observed both in mosquitoes as well as in humans. In humans, vertical transmission results in the infection of the fetus and results in fetal loss, microcephaly, and a variety of CNS disorders. Horizontal transmission can occur in many different ways. It can happen in the form of sylvatic cycles where ZIKV transmission occurs between non-human primates (NHPs) and mosquitoes, whereas in urban transmission, transmission occurs by contact of infected mosquitoes with humans. Human-to-human horizontal transmission of ZIKV occurs through body fluids, contaminated instruments and blood transfusion, etc. [76,77,78,79]. The figure was created at https://BioRender.com.

The principal mode of transmission of ZIKV is through mosquitoes; however, studies have found that various other routes may play an important role in the transmission of the disease. These include venereal transmission, vertical transmission, transmission to infants through mother milk, and blood transfusion This makes it very difficult to devise an appropriate strategy for the control of disease in countries where the disease is endemic. The transmission cycle of ZIKV is illustrated in Figure 3.

### 2.1. Vector Borne Transmission

Vector-borne transmission of ZIKV occurs through mosquitoes [13,80]. In the sylvatic transmission cycle, ZIKV circulates between non-human primates and mosquitoes, while in the urban transmission cycle, ZIKV circulates between humans and mosquitoes. The presence of ZIKV has been reported from many species of mosquitoes in Africa and Asia (*Aedes aegypti* (*Ae. aegypti*), *Ae. africanus*, *Ae. albopictus*, *Ae. apicoargenteus*, *Ae. furcifer*, *Ae. luteocephalus*, *Ae. opok*, *and Ae. vittatus*) which may transmit the virus to humans [53,81,82,83,84,85,86,87,88,89,90]. However, in Yap and French Polynesia, the transmission of ZIKV was suggested to be through *Ae. hensilli* and *Ae*. *Polynesiensis,* respectively) [91,92,93]. The extrinsic incubation period is reported to be 10 days [84]. The vector competence (the ability of the vector to transmit the virus biologically) has not been determined for ZIKV transmission in most of these species [94] and it may be the area of future research. In the urban cycle, the transmission of ZIKV is mainly through the *Ae. aegypti* and *Ae. albopictus*. ZIKV has been detected/isolated from wild *Ae. aegypti*, and they have been demonstrated to be competent vectors in the laboratory where they can transmit the virus to mice and rhesus monkeys [82,86,88]. Recent studies have demonstrated ZIKV transmission by *Ae. albopictus* as well [84,95]. Both of these mosquitoes remain active during the daytime, and they are prevalent in tropical and subtropical regions [96,97]. However, the geographical range of *Ae. albopictus* is further extended to the temperate regions [98,99,100]. In the USA, both of these mosquitoes have been found in southern and south-eastern states and their maximum numbers have been found from June to October [101].

### 2.2. Sexual Transmission

Many cases of transmission of ZIKV have been observed following sexual contact. Several studies have demonstrated ZIKV transmission from males to females. In most cases, the male had a travel history to ZIKV-endemic countries, and when he returned, his partner became infected [102,103,104]. ZIKV’s presence has been detected in human semen [105]. It has been found that ZIKV can persist in semen for over 6 months following infection, suggesting the possibility for prolonged sexual transmission [106,107,108]. Later studies demonstrated that sexual transmission from male to male is also possible [109]. ZIKV RNA has also been detected in urine and saliva [110,111]. Experiments conducted on lab animals have demonstrated that ZIKV is able to replicate in vaginal and rectal mucosa, thus serving as a reservoir of infection [112].

### 2.3. Vertical Transmission

ZIKV has been reported to be transferred from mother to fetus during the gestation period. Normally the placenta acts as an effective barrier to curtail transmission of infections from mother to fetus. However, ZIKV is able to bypass these barriers by unknown mechanisms [113]. Studies conducted in a mouse model demonstrated that ZIKV damages the placenta and results in fetal death [114]. In Brazil, during the ZIKV outbreak, a large increase in the number of neonates with microcephaly was reported from mothers who were infected with ZIKV [115]. ZIKV antigen and RNA have been detected in the placenta, amniotic fluid, and fetal brain tissue [115,116,117]. Therefore, it can be postulated that ZIKV is transmitted from mother to fetus through a damaged placenta (induced by ZIKV) and then ZIKV damages the nervous system of the fetus.

### 2.4. Venereal Transmission in Mosquitoes

The maintenance of arboviruses in nature is significantly facilitated when venereal transmission occurs alongside other modes of transmission. Venereal transmission in mosquitoes has been reported for multiple arboviruses, including ZIKV [118,119]. ZIKV is found to be transmitted in the sexual fluids of mating mosquitoes. Intrathoracically ZIKV-infected *Ae. aegypti* males can transmit the virus to uninfected females during mating, and *Ae. aegypti* females who are orally infected with ZIKV have the potential to transmit the virus to uninfected males by copulation under laboratory conditions [120]. Furthermore, *Aedes* mosquitoes acquire and transmit Zika virus by breeding in contaminated aquatic environments [121]. The continuous venereal transmission in mosquitoes could pose a significant public health concern by boosting infection rates and virus spread.

### 2.5. Transmission Through Breast Milk

Detection of ZIKV RNA has been observed in the breast milk of ZIKV-infected mothers [122]. This makes it possible for the transmission of ZIKV from the infected mother to the neonates. Studies have demonstrated the presence of ZIKV RNA in babies whose mother’s serum and milk were positive for ZIKV RNA [123]. Transmission through breast milk has also been observed for the flaviviruses WNV and DENV [124,125].

### 2.6. Transmission Through Ocular Fluid/Tears

Researchers have found the existence of ZIKV in tears thirty days following ZIKV infection. Their study suggests the possible transmission of ZIKV through tears [126,127]. This is possible as previously a case study revealed that transmission through tears occurred from mother to her child [128].

## 3. Zika Virus Associated Neurological Diseases

**Figure 4:**
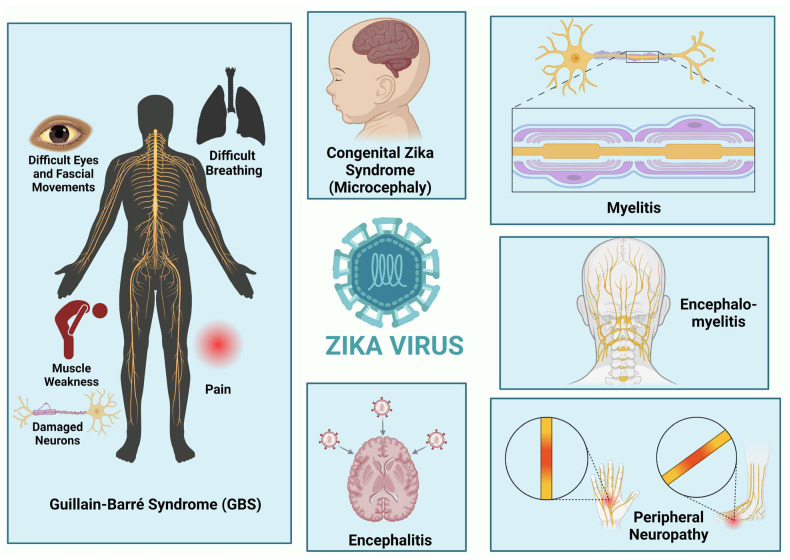
Neurological diseases associated with Zika virus infection. The figure was created at https://BioRender.com.

ZIKV infection has been reported to be associated with a number of neurological diseases (illustrated in Figure 4) which are described in detail below.

### 3.1. Microcephaly

Congenital microcephaly has been previously associated with viral infections, e.g., rubella and cytomegalovirus. However, it was unknown for ZIKV until the Brazilian outbreak [129]. ZIKV RNA was also detected in the amniotic fluid of these fetuses [130]. In 2015, Brazilian authorities reported that the incidence of microcephaly was twenty times higher compared to previous years. This led to further investigations, and in two years (2015–2016), more than 8000 cases of microcephaly were reported [131]. Later on, an analysis of an outbreak in French Polynesia also demonstrated a correlation between ZIKV and microcephaly [132,133]. Further investigations showed that aborted fetuses (during 1st and 2nd trimesters) contained ZIKV RNA in the amniotic fluid, meninges, CSF, and umbilical cord of the microcephalic newborn babies [57,134]. Several studies have been performed to identify the molecular mechanism of ZIKV-associated microcephaly. Cells associated with the BBB, such as pericytes, astrocytes, and endothelial cells, are susceptible to infection with ZIKV and their infection with ZIKV leads to upregulation of ICAM-1 and a variety of cytokines (IL-6, CCL5, and CXCL-10) that causes enhanced binding and subsequently crossing of the BBB by the immune cells. The access of immune cells to the CNS is associated with neuro-inflammation which contributed to the neuro-pathogenesis of ZIKV [135]. Neuronal progenitor cells (NPCs) exhibit high susceptibility to ZIKV and undergo apoptosis following infection [136]. During pregnancy, the immune response directed against ZIKV leads to the inflammation of the fetal–maternal interface, causing inflammation and disruption of barrier functions. This allows the recruitment of various immune cells to the fetal tissue, releasing TNF-α (tumor necrosis factor) and IFN-γ (interferon) that lead to neuroinflammation, resulting in the damage and destruction of NPCs required for the neural development of the fetus [137]. The CNS is susceptible to ZIKV infection during all stages of pregnancy, but earlier infection results in severe damage. CNS infection with ZIKV may result in impaired multiplication and migration of neurons, myelination, synaptogenesis, and even apoptosis that may contribute towards ZIKV-associated microcephaly [138]. In cells infected with ZIKV, mitofusion 2 (MFN-2) expression is reduced. Normal expression of MFN-2 is required for the fusion of mitochondria. Therefore, in ZIKV-infected cells, mitochondrial fragmentation occurs. This leads to the activation of apoptosis through a p53-mediated pathway in NPC cells [139,140]. Recently, Gladwyn-Ng et al. [141] have demonstrated the mechanism of apoptosis in neuronal cells infected by ZIKV in mouse embryos and neuronal stem cells (human), showing that ZIKV infections trigger unfolded proteins in cells that lead to activation of the ER stress response Subsequently, these cells migrate to the cortex and undergo apoptosis.

### 3.2. Ophthalmological Manifestations

ZIKV infection during pregnancy has been associated with congenital abnormalities in the fetal eye. Neural crest cells (NCCs) are required for normal development of the corneal and uveal stroma, the corneal epithelium, and the trabecular meshwork. They also play an important role in the closure of the optic fissure [142,143]. NCCs express AXL and thus exhibit susceptibility to ZIKV. Infection of NCCs with ZIKV results in viral replication resulting in loss of NCCs [144]. ZIKV infection can also disrupt the normal differentiation of the NCCs leading to defective development of the anterior eye segment leading to micropthalmia [142,145,146,147]. The loss of NCCs during ZIKV infection has also been found to disrupt closure of the optic fissure leading to iris coloboma and abnormal development of the lens zonules. Infection of NCCs with ZIKV can also lead to reduced thickening of the corneal stroma causing corneal ectasia [142]. It can also contribute towards impaired development of trabecular meshwork causing glaucoma [144,148,149,150]. Neuronal depletion during ZIKV infection can also contribute to thinning of the retinal ganglion layer [151,152]. Apoptosis of NCCs during ZIKV infection can also lead to defects in axonal development [145].

Ocular manifestations have been reported in approximately 50% of infants who have ZIKV-induced microcephaly. ZIKV infection has been reported to involve the eyes and this represents the viral ability to cross the blood–brain barrier (BBB), the blood–retinal barrier (BRB), and the blood–aqueous barrier (BAB). The first ZIKV-related eye abnormality was reported in 2016. More than sixty percent of people with ZIKV infections are reported to have conjunctivitis (the most common eye abnormality associated with ZIKV). Other abnormalities are associated with the anterior segment, the posterior segment, and the neuroophthalmic. The abnormalities associated with the anterior segment include microphthalmia, anterior uveitis (with or without raised intraocular pressure), and non-purulent conjunctivitis [153,154,155]. The posterior segment anomalies associated with ZIKV involve the retina and choroid and include maculopathy and multifocal choroiditis [153,156,157,158,159]. Neuro-ophthalmic conditions are related to the paresis of the abducens and oculomotor muscles and can cause ocular flutter, ophthalmoplegia, and papilledema [156,160,161,162,163]. ZIKV infection also causes optic nerve involvement, and this includes hypoplastic disk, enhanced cup-to-disk ratio, and nerve pallor. Viral entry into eyes is facilitated by both hematogenous and retrograde transport of ZIKV through the optic nerve and optic tract [164]. Although ocular tissue is immune privileged, it is more permissive to ZIKV infection because of the higher viral load [151]. Certain cells in the retina are relatively more permissive to ZIKV infection including RPE cells, pericytes, and endothelial cells [165]. Choriocapillaris (highly fenestrated) are highly susceptible to ZIKV infection and it can facilitate the ZIKV entry into the RPE cells, which are also permissive to ZIKV infection. RPE infection results in inflammation and this subsequently leads to breaking the BRB [108,166]. Normally, the immune response helps the host in eliminating the infection, however, in this case, immune-mediated injury to the eye tissue leads to pathogenesis of ZIKV-associated eye abnormality [167]. Infection of BRB with ZIKV leads to the generation of IL-1β, IL-6, IFN-α, IFN-β, TNF-α, CCL5 and CXCL-10. This leads to subsequent events that culminate in the atrophy of the retina and RPE mottling [168,169].

Another mechanism of viral entry in ocular tissue is through ZIKV-infected monocytes, which possess the ability to cross the retinal microvasculature endothelium and subsequently cause retinal infection. Later on, ZIKV-infected monocytes become activated and release cytokines and chemokines that result in immune-mediated injury/damage to ocular tissue [170,171]. The cornea can also act as reservoir of ZIKV infection as it expresses RIG-1, MDA-5, and TLR 3 which facilitate the synthesis of IFN-I and III to mediate antiviral response [136,172]. Infection of the cornea with ZIKV leads to the synthesis of the IL-1β, TNF-α, CCL5, and CXCL-10 which recruits other cells of the immune system to the cornea and this promotes inflammation and damage to corneal tissue leading to keratitis [173]. Through the hematogenous route, ZIKV infects the iris and ciliary body. From here, the virus can infect BAB cells, which damages this barrier and results in viral entry into the aqueous humor [133]. From the aqueous humor, ZIKV gains entry to the trabeculae meshwork and results in the production of cytokines and chemokines including IL-1β, IL-6, IL-1, IFN-α, IFN-β, CCL5, and CXCL-10. This leads to the recruitment of the other immune cells such as Th1 and CD8+ cells that secrete TNF-α and IFN-γ, damaging the trabeculae meshwork and resulting in blockage of aqueous humor outflow, leading to enhanced intraocular pressure and causing glaucoma. Persistent glaucoma can damage the optic nerve and retinal ganglion cells leading to optic neuropathy [174].

### 3.3. Guillain-Barré Syndrome (GBS)

GBS is a condition where there is immune-mediated polyradiculoneuropathy. The condition is characterized by the loss of reflexes and flaccid paralysis. The disease occurs in 1/100,000 persons. It has been observed that GBS typically occurs after certain infections such as *Campylobacter jejuni*, Epstein–Barr virus (EBV), Cytomegalovirus, and HIV. Sporadic GBS cases have also occurred following the flaviviral infections (WNV, DENV and JEV) [175]. In 2014, the first report was published about the occurrence of GBS following ZIKV infection in French Polynesia. It was reported that in ZIKV patients, 1/4000 people suffer from GBS (20 times increased incidence) [176]. In the recent ZIKV outbreak in the Americas, approximately 1500 cases were reported between 2014–2015. At the majority of locations, GBS incidence following ZIKV was reported to be 2–10 times higher than background [177]. In Colombia, more than 100 thousand cases of ZIKV were reported with 42 GBS cases/100,000 ZIKV cases. These findings indicate the possibility of the association of ZIKV with GBS [178]. Until now, no clear mechanism is known about the molecular pathogenesis of ZIKV-associated GBS. However, it is thought that the mechanism of molecular mimicry may be an important contributing factor in the pathogenesis of disease (i.e., antibodies formed against ZIKV cross-react with myelin sheath or other neural tissues). However, others have proposed that immune system dysregulation occurs (that is not directly against ZIKV) and that contributes towards its pathogenesis [176].

### 3.4. Cerebrovascular Involvement

Certain viral diseases are associated with cerebral vascular abnormalities. Recently, ZIKV cases have also been reported to involve cerebral vascular abnormalities. A 10-month-old child infected with ZIKV was admitted to hospital with ischemic stroke and had serum positive for ZIKV [179,180,181]. The exact mechanism remains to be determined; however, it is thought that higher susceptibility of endothelial cells to be infected by ZIKV is a major factor and is responsible for cerebral vascular abnormalities [182].

### 3.5. Encephalitis

ZIKV infection is considered a potential cause of encephalitis. An 81-year-old patient with a travel history to a ZIKV-affected area had reduced consciousness, limb paresis (upper right), and hemiplegia (left side). The patient was ZIKV positive by RT-PCR and viral culture and isolation. The patient was treated and survived. However, fetal cases of ZIKV-associated encephalitis have also been reported. In Brazil, a 37-year-old (y.o.) female was admitted to the hospital with a complaint of rash and joint pain. Later on, she suffered from weakness in her lower limbs, confusion, and difficulty with speech. RT-PCR was positive for ZIKV, while the serum analysis was negative. The patient later died due to brain swelling, leading to increased intracranial pressure [154]. In the Dominican Republic, a female was admitted to the hospital with complaints of neck stiffness and confusion. Her condition deteriorated, and seizures began. CSF examination revealed an increased number of WBCs (white blood cells), the majority of which were lymphocytes (80%). RT-PCR of urine was positive for ZIKV RNA (but negative in the CSF and serum). However, IgM antibodies against ZIKV were detected from CSF and serum, confirming ZIKV exposure. MRI examination revealed cortical edema, a common finding in meningoencephalitis [163]. In another study on ZIKV, subcortical hemorrhage and bilateral lesions were observed in the temporal and frontal regions [162].

### 3.6. Myelitis and Encephalomyelitis

ZIKV infection has been associated with myelitis (inflammation of the spinal cord) and encephalomyelitis (inflammation of the brain and spinal cord), which can result in reduced consciousness, urinary retention, and paresis of lower limbs [183]. The first case of myelitis associated with ZIKV was reported in Guadeloupe in a girl (15 y.o.) who was admitted to the hospital with a complaint of headache and conjunctivitis. Later, her condition continued to deteriorate, and she suffered from left-sided weakness and urinary retention. MRI examination revealed a normal brain, whereas abnormalities were revealed in the spinal cord cervical area and thoracic region. CSF, urine, and serum were found to be positive for ZIKV by RT-PCR [184]. In another study, a 26 y.o. Brazilian male was admitted to the hospital with a major complaint of fever, malaise, and neck stiffness, CSF and urine examination revealed that the patient had a ZIKV infection. CSF analysis showed hyper-protein levels and 100 WBCs (white blood cells) per mL (95% mononuclear cells) [183]. Other studies have also indicated an association between ZIKV and myelitis/encephalomyelitis [183,184,185].

## 4. Zika Virus Infection and Extracellular Vesicles

Viruses can employ extracellular vesicles (EVs) that can facilitate infection, pathogenesis, and transmission. EVs are particles surrounded by membranes that can be released into the extracellular space by living or dying cells. Various viral components may be packed inside the EVs [186]. One of the final fates of EVs is their release into blood circulation, through which they can reach distant cells and tissues [187]. It is important to note that EVs share most of the biochemical and biophysical properties of viruses. Both EVs and viruses (naked and enveloped) use the Endosomal Sorting Complex Required For Transport (ESCRT) complex [188,189]. Moreover, Rab GTPases (particularly Rab11, Rab35, and Rab27A/B) are also shared during the viral replication cycle as well as EV biogenesis [190]. Many studies have indicated that EVs play a facilitating role during viral infections. These effects can be due to EVs carrying viral receptors, carrying viral particles themselves, or carrying their genome, proteins, or miRNA. Ultimately, it can lead to the priming of the recipient cells and/or inhibiting IFN pathways, apoptosis of immune cells, and the transmission of viral genome and/or proteins to distant organs and tissues [188,191,192,193]. In the case of ZIKV, NS1 protein, viral E protein (inside and outside) and viral RNA can be packed inside the EVs [194]. Interestingly, mosquito cells (C6/36 cells) infected with ZIKV are found to release EVs (containing viral RNA and protein E), which was found to infect both naïve mosquito and mammalian cells (monocytes and endothelial cells) [195].

Given ZIKV’s neurotropic nature, it is important to emphasize that ZIKV-derived EVs are found to disrupt the structure of human brain microvasculature endothelial cell junctions (hcMEC/D3), facilitating ZIKV entry into cells of the human CNS [196]. ZIKV can also spread to the CNS through autophagy (a process associated with viral infections and may be regulated by EVs [197,198,199]. In the case of ZIKV, the autophagic vesicles may play an important role in crossing the placental barrier. Normally, the placenta is protected from various pathogens (aided by the autophagy process); however, ZIKV can hijack this process [200,201]. A study using a mouse model has shown that chloroquine or hydroxychloroquine inhibits viral replication (autophagy-dependent) and also effectively prevents mother–fetal transmission of the virus [202,203].

## 5. Models for Studying Zika Virus Infection and Pathogenesis

Developing appropriate cellular and animal models for studying ZIKV is becoming increasingly important to gain appropriate understanding of viral pathogenesis and host-related factors that aid or inhibit viral replication and to devise appropriate disease prevention and treatment strategies.

### 5.1. Cell Lines

**Figure 5:**
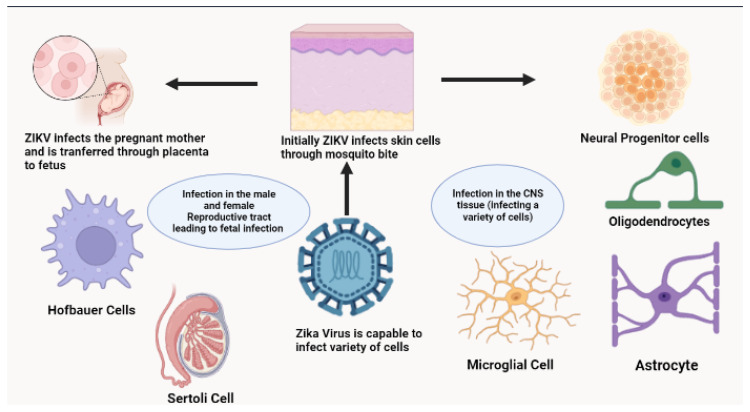
Cells susceptible to Zika Virus infection: ZIKV gains access to body through bite of an arthropod vector. Following bite, viremia occurs and in a pregnant mother, ZIKV infects the Hofbauer cells in placenta and in this way, it is transmitted to the fetus. In fetus, ZIKV is capable of infecting a myriad of cells, including neural progenitor cells, oligodendrocytes, astrocytes, and microglial cells. This may result in the causation of several congenital neural diseases as discussed in the paper. Furthermore, ZIKV has also been found to establish active infection in male reproductive tract and thus, is transmitted from male to female [204,205,206,207]. The Figure was created at https://BioRender.com.

The cell lines are widely used to grow and study ZIKV because they provide a controlled environment for viral replication. These cell lines allow scientists to watch viral behavior, examine molecular interactions between ZIKV and host cells, and test antiviral agents. The use of cell lines is essential to virology as they provide a reliable, reproducible system for studying viral pathogenicity and mechanisms in a laboratory setting. The cells susceptible to Zika Virus infection are illustrated in Figure 5 and detailed comprehensively in Table 2.

### 5.2. Organoids

Although cell culture has been used to determine ZIKV replication and pathogenesis, this system is not without its downsides. Cells are unable to mimic in vivo conditions as they lack the cellular structure, composition, and physiology of an organ in which a virus replicates [222]. To overcome this drawback, people are now developing organ analogues in vitro where stem cells are used to generate 3D tissue structures resembling organs. These 3D models are known as organoids and are being used to model and better understand the molecular events that occur following viral invasion and replication in that organ [223]. Studies have provided confirmatory evidence of microcephaly following ZIKV infection by using organoids [224,225]. Qian et al. (2016) used a mini-bioreactor to generate brain organoids and demonstrated that ZIKV infection in brain organoids that mimic the first-trimester fetal brain resulted in ZIKV infection of NPCs, IPC, astrocytes, and neurons, while infection of brain organoids mimicking the second-trimester fetal brain exhibited ZIKV infection of the outer radial glial cells [224]. These findings are correlated with reduced cellular proliferation and enhanced apoptosis providing evidence of ZIKV-associated microcephaly [136]. Yoon et al. showed that the ZIKV NS2A protein is responsible for the alteration and reduced multiplication of the radial glial cells resulting in altered neuronal positioning in the brain [226]. Another study reported that ZIKV infection of NPCs during early pregnancy was associated with the upregulation of TLR-3 receptors, leading to apoptosis of NPCs, resulting in microcephaly [227]. Recently, brain organoid models have also been used to determine the various drug targets against ZIKV [228,229,230,231]. Organoids resembling the placenta have also been developed using first-trimester human chorionic placental villi where ZIKV established infection and proliferated in Hofbauer cells, villus cytotrophoblasts (CTB), and produced the NS3 and E protein [232,233]. To summarize, organoids offer a promising approach to studying the ZIKV viral pathogenesis and also to screening compounds’ antiviral activity in a system more relevant than in vitro cell culture.

### 5.3. Animal Models

Animal models have been developed to study ZIKV replication and pathogenesis in vivo.

#### 5.3.1. Non-Human Primates (NHPs)

Non-Human Primates (NHPs) exhibit significant similarities with humans (pregnancy physiology, long gestation periods, and hemomonochorial placenta) and offer a promising model to study ZIKV infection, neuropathogenesis, and drug discovery. They also exhibit vertical transmission from mother to fetus similar to humans [234]. Rhesus macaques, olive baboons, marmosets, and pigtail macaques have been employed to study ZIKV pathogenesis during gestation. Olive baboons have been able to demonstrate clinical infection when infected during the gestation period. When infected during 4th month of pregnancy, they developed conjunctivitis and moderate rash, with viremia peak at 7 dpi (days post-infection). Pro-inflammatory cytokines and immunoglobulins were also detected [235,236]. In other NHPs (i.e., rhesus macaques, marmosets, and pigtail macaques) no clinical infection occurs following ZIKV inoculation. However, in all NHPs, antibodies are formed following ZIKV infection, and placental and fetal ZIKV infection is observed. In rhesus macaques, fetal loss occurs following ZIKV infection, while marmosets and olive baboons exhibit viral RNA in amniotic fluid and placenta. Severe placental infection is observed during pregnancy in marmosets and rhesus macaques [237,238,239].

#### 5.3.2. Mice

Many viral replication and pathogenesis studies involve the use of mice as animal models because they have large litter sizes and a short gestational period. However, variability in their placental structure and heterogeneity in gestation can be problematic [240]. Another complication occurs due to differences in the cell biology of mice as compared to humans. On viral entry, the viral NS5 protein is able to degrade the cellular STAT2 protein, thereby making IFN signaling ineffective, resulting in viral replication and the development of clinical disease. Degradation of STAT-2 is not observed in mouse cells infected with a virus, leading to unimpaired IFN signaling resulting in low viral titer and failure to develop clinical infection [241]. Therefore, biological modifications are needed to use mice for studying ZIKV replication and pathogenesis events. This can be achieved by using either immunocompromised mice or using alternative routes of inoculation.

##### Immunocompromised Mice

Immunocompromised mice are modified for interferon (IFN) insufficiency. This may be conducted by either creating knockout (KO) mice, e.g., interferon alpha receptor knockout (Ifnar-/-), or by using antibodies to block the IFN receptors (MARI-5A3) [242,243]. It has been found that pregnant Ifnar-/- mice exhibit high susceptibility to ZIKV infection with subcutaneous inoculation and are able to produce fetal and placental abnormalities [114]. The infection is established in homozygous as well as heterozygous mice, suggesting that partial deficiency in IFN signaling is sufficient to produce fetal neuropathology. As ZIKV infection can occur through venereal transmission; therefore, models have been developed for this purpose. Intravaginal inoculation of ZIKV from 5–8 days of conception results in high levels of viral RNAs in the placenta and fetal tissues in Ifnar-/- mice [244]. ZIKV infection (intravaginal) of IFN regulator factor (IRF) KO e.g., Irf3-/- Irf7-/- mice exhibit higher vaginal and fetal titers of the virus. This implies that inhibition of IFN signaling is a key factor in establishing different mice models.

##### Immunocompetent Mice

As discussed previously, ZIKV infection often fails in immunocompetent mice. However, some researchers have used alternative strategies to produce ZIKV infection by using higher viral titer or other routes of inoculation. A Brazilian ZIKV strain has been used to produce successful fetal infection in SJL mice. In this study, high viral inoculation (10^10^ to 10^12^ pfu/mL) was used to produce uterine and fetal infection that ultimately resulted in fetal microcephaly [245]. However, these mice showed higher levels of T cells in the blood and later on developed autoimmunity and sarcoma [246]. The intraamniotic route has also been used for ZIKV inoculation in embryos and resulted in damage to the retina and blood–brain barrier and produced neuronal death and defects in motor neurons in the fetal brain. Intrauterine infection also resulted in ZIKV infection of placental endothelial and trophoblast cells and in the fetus, the virus localizes in the microglial, endothelial, and NPCs of the fetal brain [67,247,248]. Another model has also been developed to study the postnatal CZS progression by inoculating C57BL/6 mice intraperitoneally, and it was seen that the fetus had reduced body weight and microcephaly. Later on, these mice exhibited motor and memory deterioration in adulthood [249]. These studies pave a path towards viral replication and neuropathogenesis studies in immunocompetent mice models that may be deployed for further understanding of ZIKV neuropathogenesis and drug discovery.

#### 5.3.3. Farm Animals

Although less common as animal models, pregnant sheep, and pigs may be used as a model for ZIKV replication and pathogenesis. They are widely available and exhibit susceptibility to the virus [250,251]. They can also be used as a model for human pregnancy and fetal growth [252]. ZIKV infection during the first trimester in sheep resulted in placental pathology, reduced fetal growth, and fetal loss [253]. Maternal ZIKV infection during the 4th to 5th week resulted in the detection of ZIKV RNA in the PBMCs of the mother; however, clinical infection failed to occur [253]. Similarly, in pigs, intrauterine infection during pregnancy resulted in placental abnormalities as well as microcephaly in piglets [254]. It has also been seen that following intrauterine ZIKV infection, the African ZIKV lineage was found to produce a more pronounced viral infection as compared to the Asian strain [255]. These findings clearly establish the role of sheep as a possible animal model to study fetal neuropathogenesis due to ZIKV.

#### 5.3.4. Rats

As rats become immunocompromised during the final stage of gestation, similar to humans, they may also be utilized as a model to investigate ZIKV neuropathogenesis [256]. Upon subcutaneous (S/C) inoculation of ZIKV to pregnant rat females, reduced cortical and hippocampus volumes were observed in rats, although no pathological findings and viral detection were observed in the maternal counterpart [114,257].

#### 5.3.5. Hamsters

Infection of immunocompetent females during pregnancy was unable to produce productive viral infection; ZIKV RNA was not found in fetal or placental tissue and fetus size remained unaffected [114]. However, ZIKV infection (Malaysia Strain) in immunocompromised hamsters (8 days post-mating) resulted in ZIKV infection of placental tissue and also caused vertical transmission of the ZIKV from mother to offspring [258].

#### 5.3.6. Chicken

Chicken embryos have been demonstrated to support ZIKV viral growth in the chicken brain and other tissues [259]. Intraamniotic inoculation of ZIKV resulted in stunted embryo growth and microcephaly [260]. Furthermore, comparison of the Asian and African ZIKV strains showed that the African strain caused more embryonic pathologies [261].

## 6. Treatment and Prevention Against Zika Virus and Zika Virus Associated Neuropathies

### 6.1. Prevention from Zika Virus Through Vaccines

One of the major methods for viral disease prevention and control is vaccination. Immediately after the outbreak of ZIKV, research began to find ideal vaccine candidates to prevent future ZIKV outbreaks [262]. Several vaccine candidates have emerged; however, very few have been used in clinical trials. The various ZIKV vaccine candidates are summarized in Table 3.

#### 6.1.1. Purified Inactivated Zika Virus Vaccines (PIZIKV)

Several inactivated vaccines have been developed and tested by animal models. The majority of these vaccines have been able to demonstrate higher antibody titer, reduced viremia, successful passive transfer of antibodies, and protection in mice (interferon deficient) after homologous or heterologous vaccination [263]. In India, one vaccine has entered phase I clinical trials (CTRI/2017/05/008539) [291]. Using mice and non-human primates (NHPs), an aluminum hydroxide-based inactivated ZIKV vaccine has demonstrated promising antibody titers and protection against homologous antigen challenge [265]. Another PIZIKV provided complete protection in a dose-dependent manner (0.016 to 10 ug) and was found to be effective one year following vaccination in macaques [266]. A double-blind randomized controlled trial (Phase I) was performed to evaluate the safety and immunogenicity of PIZIKV, and it was found that the vaccine was safe for 52 h. However, protection was not found to be effective as only a single dose was given and for better immunogenicity, a double dose may be needed [268].

#### 6.1.2. Live Attenuated Vaccines:

Live-attenuated vaccines (LAVs) are known for eliciting swift and long-lasting protective immunity and represent a crucial strategy for managing flavivirus diseases [292]. These are made by modifying the virus in such a way that it may replicate within the organism and stimulate the immune system without causing disease. Since 1938, they have been used as the gold standard as they exhibit good safety and effective protection from viral infection [293]. LAVs are known to stimulate the immune system for long-lasting periods (without the need for an adjuvant or any booster). Currently, three approaches are being used for generating ZIKV LAVs, which are (i) generating chimeric strains (using the backbone of an attenuated flavivirus), (ii) codon deoptimization, and (iii) mutagenesis. One of the most important advantages of using live vaccines is their ability to elicit robust humoral and cell-mediated immune responses (with a single dose of immunization); as the virus continues to multiply within the host cells, allowing continuous loading of MHC (I and II) with viral peptides. In addition, they are cost-effective (both during manufacturing as well as transportation). The past experiences of successful development of LAV against Japanese Encephalitis Virus (JEV) and Yellow Fever Virus (YFV) have clearly shown their potential to be used as safe viral vaccines [279,293]. However, manufacturing of live vaccines usually requires eggs or cell culture and also maintaining a cold chain during transportation. In countries having warm climates, maintaining cold chain only can account for up to 80% of the total vaccine cost [294]. Another major concern associated with the use of live vaccination is inadequate attenuation (once they are at the stage of the human clinical trials [295]. A variety of LAVs have been tested against the ZIKV [292], and the selective studies are summarized in Table 3.

#### 6.1.3. Nucleic Acid Vaccines

DNA and RNA-based vaccines are being developed for different infectious diseases. They offer the advantage of prolonging immune response following vaccination and induce both humoral and cell-mediated immunity (CMI) [296]. However, one major disadvantage is that DNA vaccines often fail to elicit adequate immune response or offer protection from challenge [297]. Despite these advantages, DNA vaccines may have the risk of eliciting autoimmunity (by eliciting anti-DNA antibody production) and also causing insertional mutations [298]. Another point to keep in mind is that in the case of DNA vaccines, DNA must enter inside the nucleus of the cell, and then after transcription, mRNA must travel outside the nucleus (into the cytoplasm), where it is translated into the proteins. In contrast, for RNA vaccines, RNA can directly be translated into proteins in the cytoplasm, which are then secreted from the cells (into the extracellular matrix) where they can be presented to the antigen-presenting cells (APCs). mRNA vaccines have become more popular after the introduction of SARS-CoV-2 vaccines. However, it is also important to note that storage of mRNA vaccine requires maintaining a cold chain, which may not be feasible in developing countries [299]. The summary of various DNA vaccines available against ZIKV is summarized in Table 3.

#### 6.1.4. Subunit Vaccines

Due to their rapid production and stability, a variety of ZIKV vaccines have been developed. The majority of these vaccines have used the Envelope protein (E protein) and its different segments. These vaccines have been tested in various animal models, which are summarized in Table 3.

#### 6.1.5. Viral Vector-Based Vaccines

Viral vector-based vaccines are prepared by using a vector virus for the insertion of the genetic components of ZIKV, and they have been found to induce good humoral immunity. Lentovirus, adenovirus, and retroviruses are known to be good viral vectors. Various ZIKV vaccines have been developed using various viral vector systems, which have been summarized in Table 3.

### 6.2. Zika Virus Prevention Through Vector Control

Mosquitoes play a central role in the transmission of ZIKV. Therefore, reducing ZIKV transmission through vector control methods involves diverse and innovative strategies and tools designed to manage disease-spreading mosquito population. One of the most widely used strategies is the use of chemical insecticides (due to their cost-effectiveness and easy availability). However, due to the emergence of insecticide resistance and their deleterious effects on the environment, the focus is now diverted towards various other control methods [300,301]. Due to their eco-friendly nature, herbal-based control methods have now gained attention. Promising insecticide activities have been shown by extracts derived from the *Illicium verum*, *Zanthoxylum limonella*, *Cymbopogon citratus*, *Cymbopogon winterianus*, *Eucalyptus citriodora*, *Eucalyptus camaldulensis*, etc. [302,303,304,305,306,307,308]. Furthermore, nanoparticles derived from *Ambrosia arborescens* have also shown good larvicidal activity. However, their toxicity profile should be determined against other environment-friendly insects, animals, and humans [307]. Similarly, biological control methods are also being employed due to their minimal impact on the environment [309]. This includes the use of entomopathogenic bacteria and fungi. Various species of entomopathogenic fungi (*Metarhizium anisopliae*, *Beauveria bassiana*, *Aspergillus flavus*, *Aspergillus fumigatus*, and *Aspergillus terreus*) and bacteria (*Bacillus thuringiensis israelensis* (Bti), *Wolbachia*) have been tested against ZIKV-transmitting mosquitoes [310,311,312,313,314,315]. Particularly, the introduction of *Wolbachia* in mosquitoes has demonstrated the ability to disrupt the reproductive potential of disease-transmitting vectors. Similarly, the use of various natural predators, i.e., Toxorhynchites mosquitoes, fishes, tadpoles, and copepods, have also been used against the mosquitoes and have shown varying degrees of efficacy in controlling the mosquito population [316]. Moreover, genetic engineering principles have been applied to release genetically modified mosquitoes into the environment [317]. Furthermore, the use of the sterile insect technique is also an innovative approach to control the mosquito population [318]. When used in combination, these strategies offer an integrated approach to vector control, addressing both short and long-term goals of reducing ZIKV and its induced neuropathies. A summary of vector control strategies developed so far is provided in Table 4.

### 6.3. Treatment for Zika Virus

#### 6.3.1. Antivirals Against Zika Virus

Since the outbreak of ZIKV, many compounds have been proposed to possess antiviral activity against ZIKV. However, in the majority of cases, treatment is symptomatic, and no specific antiviral has been approved for ZIKV treatment. A myriad of approaches are being adopted for searching for anti-ZIKV drugs. These strategies include the screening of different compounds for their anti-ZIKV activity or through repurposing drugs that are already being used for the treatment of other viral infections. In addition, the anti-ZIKV activity of several herbal/plant extracts is also being evaluated. Broadly, anti-ZIKV compounds have been divided into 2 major categories: (a) direct-acting antivirals, which inhibit the viral replication cycle; (b) antivirals targeting host cells [75]; both of these are summarized in Table 5. The former drugs act on the viral RNA-dependent RNA polymerase (RdRp); NS5. Polymerase inhibitors and nucleoside analogs are included in this category. They act by targeting the catalytic domain of NS5. The other category of drugs (host-targeting antivirals) targets the host cell processes (that play an important role in viral replication). Viruses need host nucleosides for their genome replication. The host-acting antivirals can act on various steps of viral replication (host cell binding, entry, replication complex formation, viral maturation, and release from the host cell). An advantage of targeting host processes is that they are less susceptible to developing drug resistance [75].

#### 6.3.2. Medicinal Plants as Zika Virus Antivirals

Modern scientific research has found numerous synthetic antiviral medications that are effective against a variety of viral infectious disorders throughout the past few decades. Unfortunately, a wide range of negative consequences associated with these synthetic medications have been documented. They may occasionally lose their potency against newly arising virus resistance strains [381]. Furthermore, people in developing nations cannot afford expensive synthetic medications for the treatment of viral infections. Medicinal plants may offer economical and efficient antiviral medications. To date, no plant-derived antiviral has been approved for clinical use against ZIKV. Various research has been conducted to identify medicinal plants possessing anti-ZIKV activity, and they have been summarized in Table 6.

## 7. Zika Virus and Its Potential Oncolytic Activities

ZIKV exhibits tropism for a variety of cell types including normal neural stem/progenitor cells (NSCs/NSPs), microglial cells, astrocytes, and oligodendrocyte precursor cells [406,407,408]. Following the entry into cells, the virus disrupts growth and development, causing cell death. Studies in various animals have also supported the association between ZIKV infection and neurodegeneration. Inoculation of ZIKV in rhesus monkeys during early pregnancy has been shown to result in the aberrations of the microglial cells and also cortical plate thinning (3 weeks post-inoculation) [409,410]. Therefore, ZIKV is a promising virus that may be used as an oncolytic virus against glioblastoma as it infects the tumor cells. Particularly, in vitro studies have shown that the ZIKV NS5 protein inhibits the migration, proliferation, and invasion of glioblastoma. Studies in mice have shown that it increases the survival of C57/B6 mice with intracranial glioblastoma [411]. Another study in C57/B6 mice has shown that ZIKV increases the survival of mice with glioblastoma (with the suppression of tumor volume as well as the absence of clinical signs) [412]. Further molecular studies have revealed that ZIKV protease causes the cleavage of human cellular gasdermin D (GSDMD), which causes the activation of pyroptosis (caspase-independent) [413]. ZIKV infection in immunocompetent dogs (suffering from glioblastoma) has also been shown to decrease the tumor mass volume and ameliorate neurological signs. Further investigations revealed an increase in the number of immune cell infiltration in the tumor microenvironment [414]. This increase in the numbers of immune cells (CD4+ and CD8+ T cells) has also been observed in C57BL6/J mouse models (suffering from glioblastoma) infected with ZIKV. This infiltration of T cells into the tumor microenvironment provides enhanced protection to mice. ZIKV infection is associated with enhanced activation of the type I interferon signaling pathway in glioblastoma cells and increases the glioblastoma sensitivity to the PD-L1 blockade, which is an important immune checkpoint [415,416,417].

## 8. Future Perspectives in Zika Virus Control

Since its emergence in 1947, ZIKV has only caused widespread epidemics in the last two decades. The outbreaks of ZIKV also suggest the dynamics of transmission variability of ZIKV. The outbreak of ZIKV in the Americas caused a rapid increase in studies of ZIKV pathogenesis and its control measures and treatment strategies. It is of prime importance to continue working on establishing virological and immunological diagnostics kits for ZIKV with enhanced specificity and sensitivity. Another area of focus is ZIKV pathogenesis. Finally, prevention and treatment strategies remain pivotal to curtail ZIKV-related morbidity and mortality. Currently, many vaccines are being tested on animal models, but very few have entered into clinical trials, there is a dire need to improve the quality of good vaccine candidates. Plus, the development of genetically modified mosquitoes could be a groundbreaking tool for controlling Zika virus transmission.

## Figures and Tables

**Table 1:** Comprehensive overview of Neurotropic Flaviviruses.

Type of Flavivirus	Geographical Distribution	Vectors	Vertebrates Hosts	Incubation Period	Neurological Complications	Diagnosis	References
West Nile Virus (WNV)	Africa, Asia, Middle East, United States (1999) and France (2002)	*Culex pipiens* and *Culex quinquefasciatus* (America, Asia, Africa) *Culex australicus,* and *Culex globcoxitus* (Australia).	Wild birds, birds from corvidae family (crows, magpies, jays) and horses	4 to 14 days	Ataxia, extrapyramidal syndrome, confusion, cranial neuropathies, encephalitis, meningitis, somnolence, flaccid paralysis (asymmetric), and coma.	ELISA (IgM; serum and spinal fluid), RT-PCR/PCR (whole blood, plasma, serum, CSF, urine, and mosquitoes), and MRI (periventricular inflammation)	[23,24,25,26,27,28]
Japanese Encephalitis Virus (JEV)	Asia, Western Pacific Island	*Aedes albopictus*, *Culex pipiens*, *Culex tritaeniorhyncus*, *Culex quinquefasciatus,* and others.	Pigs and water birds	5 to 15 days	Seizures, neurological abnormalities (focal), meningitis, and encephalitis.	MRI, ELISA, VNT/PRNT, HI, CFT, and RT-PCR.	[16,29,30]
Zika Virus (ZIKV)	South and Central America, Asia, Africa, and Caribbean	*Aedes aegypti*, *Aedes albopictus*, *Aedes vexans*, *Aedes vittatus,* and *Culex quinquefasciatus.*	Monkeys (Rhesus macaque)	7 to 14 days	Associated with the onset of GBS and microcephaly hypoplasia of the spinal cord and brain stem.	RT-PCR, PRNT, and ELISA (IgM).	[31,32,33,34]
Saint Louis EncephalitisVirus (SLEV)	USA, Canada, Central America and Caribbean Islands.	*Culex tarsalis*, *Culex pipiens,* and *Culex quinquefasciatus*	Wild birds	5 to 15 days	Confusion, dizziness, disorientation, tremors, stiff neck, encephalitis, meningitis, and meningoencephalitis.	RT-PCR and ELISA (IgG and IgM) and IFA	[35,36,37,38,39]
WNV-Kunjin virus	Northern Australia	*Culex annulirostris*	Water birds	5 to 15 days	Confusion, drowsiness, seizures, and encephalitis.	RT-PCR	[40,41]
Murray Valley Encephalitis Virus (MVE)	Australia (Northern) and New Guinea	*Culex annulirostris* *Aedes normanensis*	Large water birds (e.g., egrets)	5 to 15 days	Flaccid paralysis, coma, confusion, drowsiness, seizures, cranial nerve palsy, tremors, encephalitis, and meningoencephalitis.	RT-PCR	[42,43,44]
Tick-borne Encephalitis Virus (TBEV)	Europe and Asia	*Ixodes ricinus*, *Ixodes persulcatus, and Ixodes ovatus.*	Birds and small mammals	7 to 14 days	Encephalitis (sub-acute to severe), meningitis, and myelitis.	RT-PCR and ELISA (IgM)	[45,46,47]
Powassan Virus (POWV)	North America and Eastern Russia	*Ixodes cookie*, *Ixodes marxi*, *Ixodes scapularis*, *Dermacentor anders oni*, *Dermacentor silvarum, and Haemaphysalis longicornis.*	Wild animals	7 to 14 days	Ataxia, seizures, mental status alternation, cranial nerve palsies, meningitis encephalitis with meningismus, and cerebral edema.	RT-PCR, ELISA (IgM), and Immunohistochemistry.	[48,49,50,51,52]

Abbreviations: ELISA (Enzyme-Linked Immunosorbent Assay), IgM (Immunoglobulin M), IgG (Immunoglobulin G), RT (Reverse Transcription), PCR (Polymerase Chain Reaction), CSF (Cerebrospinal Fluid), MRI (Magnetic Resonance Imaging), VNT (Virus Neutralization Test), PRNT (Plaque Reduction Neutralization Test), CFT (Complement Fixation Test), HI (Hemagglutination Inhibition Test), and IFA (Immunofluorescence Assay).

**Table 2:** Cellular models for studying ZIKV infection and pathogenesis.

Sr No.	Cell Line Susceptible to ZIKV		Source	Mechanistic Evidence of Infection Susceptibility	References
1	Neuronal cell lines	SF268	Human	Viral titers, synthesis of viral proteins, and cytopathic effects (CPE)	[208]
		CRL-2267, CCL-127, CRL-2271 (male origin) CRL-2266, and CRL-2149 (female origin)	Human	Viral titers and cytopathic effects (CPE)	[209]
		SH-SY5Y	Human	Viral titers and cytopathic effects (CPE)	[210,211,212]
		U87-MG	Human	--	[213]
		SK-N-SH	Human	--	[214]
2	Fibroblasts (dermal, lungs)		Human	Viral titers, synthesis of viral proteins, replication of positive and negative strands of viral genome, and cytopathic effects (CPE)	[215,216]
3	Vero cells		Non-human	Viral titers and cytopathic effects (CPE)	[212,216,217,218,219]
4	Human hepatocellular carcinoma cells (HuH-7)		Human	Viral titers and cytopathic effects (CPE)	[208,212,216,217]
5	Human cervical adenocarcinoma cells (HeLa)		Human	Viral titers and cytopathic effects (CPE)	[216,217,220]
6	A549 cells		Human	Viral titers and cytopathic effects (CPE)	[211,212,218,219,221]
**Other cell lines**					
7	Primary human amnion epithelial cells (HAECs) form the lining of the amniotic sac		Human		[217]
8	Placental (JEG-3), muscle (RD), retinal (ARPE19), pulmonary (Hep-2 and HFL), colonic (Caco-2),		Human and non-human	Viral titers, synthesis of viral proteins, and cytopathic effects (CPE)	[208]
9	Sertoli, Hs1.Tes, SEM-1 and TCam-2 cells,		Human	Viral titers, synthesis of viral proteins, and cytopathic effects (CPE)	[214]
10	Human renal carcinoma cells (Caki), Human astroglia (SVG p12, American Type Culture Collection CRL-8621 (caco-2) and Human embryonic kidney cells (293T)		Human	Viral titers, synthesis of viral proteins, and replication of positive and negative strands of viral genome	[216]

**Table 3:** Vaccines developed and tested against Zika Virus (ZIKV).

Purified Inactivated ZIKV Vaccines (PIZIKV)						
Zika Virus Strain	Vaccine Type	Development Strategy	Study Conducted	Mechanism	Route	Reference
ZIKV strain PRVABC59 (Asian genotype associated with American outbreak)	Alum-adjuvanted Inactivated	Virus inactivation with formalin followed by use of adjuvant (aluminum hydroxide).	CD-1 and AG-129 mice were vaccinated with alum adjuvanted vaccine (2 doses of vaccines having 0.1 ug of Ag). Following vaccination, AG-129 showed protection and were found to neutralize ZIKV from both African and Asian lineages.	Neutralizing antibodies may play an important role in protection when challenged with virus.	Intramuscular (IM)	[263]
ZIKV strain PRVABC59	ZIKV purified inactivated vaccine (ZPIV), adjuvanted with aluminum hydroxide	Virus inactivation with formalin followed by use of adjuvant (aluminum hydroxide).	Sanofi Pasteur Institute upgraded the vaccine and their studies in mice (BALB/c) and found that vaccine protects against homologous challenge. Furthermore, studies in cynomolgus macaques have shown that vaccines provide good humoral and CMI and also completely protect it from ZIKV challenge	neutralizing antibodies, specific T-, and memory B-cells	Intramuscular (IM)	[264,265]
ZIKV PRVABC59	Inactivated Vaccine	Aluminum hydroxide adjuvanted purified inactivated Zika vaccine	Indian rhesus macaques were vaccinated twice with ZIKV vaccine and were challenged at 6 weeks and 1 year following vaccination. It was observed that following challenge, vaccine provided protection in a dose dependent manner. Highest protection was achieved at 10 μg.	Vaccine induced neutralizing antibodies in a dose-dependent manner	Intramuscular (IM)	[266]
ZIKV PIZV in adengue virus (DENV)	Inactivated Vaccine	Purified inactivated Zika vaccine.	Two doses of vaccine protected the rhesus macaques 1 year following vaccination	Vaccine induced neutralizing antibodies	Intramuscular (IM)	[267]
ZIKV PRVABC59	Inactivated Vaccine	5 μg ZPIV and 500 μg aluminium hydroxide adjuvant (Alhydrogel).	Phase I clinical trials was conducted in humans (18–50 years of age). Computer generated randomization was used within groups and were vaccinated with 5 ug of inactivated ZIKV vaccine. Vaccine was found to be safe however, one dose was proven to be insufficient to provide complete protection and hence twice vaccination was recommended	Vaccine induced neutralizing antibodies	Intramuscular (IM)	[268]
**Live Attenuated Vaccines (LAVs)**						
**Zika Virus** **Strain**	**Vaccine Type**	**Development Strategy**	**Study Conducted**	**Mechanism**	**Route**	**Reference**
ZIKV Cambodian strain (FSS13025) and Puerto Rico strain (PRVABC59)	Live attenuated vaccine (LAV)	Nucleotide deletion from 3′ end.	The vaccine was used in IFN deficient mice (AG129). A total of 10 nts were deleted from 3′ end of ZIKV and inoculation was made in 3 weeks old mice through SC route. that ZIKV proved safe, immunogenic, and induced higher antibody titers. The vaccine also prevented the viremia following challenge (Cambodian strain FSS13025 and Puerto Rico strain PRVABC59). The attenuated virus also proved to be the non-virulent for 1 day old mice @1 × 10^4^. The LAV was also unable to be transmitted to mosquitoes.	Induction of humoral immunity and cell-mediated immunity.	Subcutaneous (SC)	[269]
ZIKV Cambodian strain (FSS13025) and Puerto Rico strain (PRVABC59)	Live attenuated vaccine (LAV)	Nucleotide deletion from 3′ end.	LAV was prepared by deletion of 20 nts from the 3′UTR of ZIKV. On challenge (with Cambodian strain FSS13025 and Puerto Rico strain PRVABC59 at 6 days post vaccination), this vaccine prevented the ZIKV infection in maternal, placental, and fetal tissues in A129 mice. In males, testes damage was prevented. Furthermore, vaccination in rhesus macaques demonstrated the effective development of humoral immune response and also prevented the infection on challenge.	Induction of humoral immunity and cell-mediated immunity.	Subcutaneous (SC)	[270]
ZIKV Puerto Rico strain (PRVABC59)	Live attenuated vaccine (LAV)	Containing NS1 gene without glycosylation, through changes in 10 nucleotides.	Vaccine was inoculated in IFN deficient mice (A129) @10^5^ PFU through SC route. Following vaccination animals were challenged with 10^6^ PFU of ZIKV PRVABC59 (heterologous strain) at 6 days of conception. At 13 days post conception, animals were sacrificed and they demonstrated minimal levels of viral RNA in maternal, placenta, and fetus, thus inhibiting the maternal–fetal transmission of the ZIKV.	Induction of humoral immunity and cell mediated Immunity CD4+ and CD8+ T cell responses.	Subcutaneous (SC)	[271]
VacDZ	Live attenuated vaccine (LAV)	Chimeric dengue/Zika virus	VacDZ was administered @10^6^ PFU per mouse (AG129 mice) for initial and booster dose (4 weeks apart). After 4 weeks of the booster dose (8 weeks after initial vaccine administration), vaccinated mice were sacrificed to determine the immune responses. Similarly, vaccinated mice were challenged with the lethal dose of ZIKV (10^5^ PFU per mouse), 4 weeks after vaccination.	Strong humoral and Th1 immune response in AG129 mice (Th1 response was stronger than Th2); also protected mice against the lethal ZIKV challenge.	Intraperitoneal (IP)	[272]
DNA-launched LAV	Live attenuated vaccine (LAV)	Plasmid-Launched LAV	Six-week-old mice (A129) inoculated with 20 µL of DNA solution. At various time points, inoculated mice were bled via retro-orbital sinus (to determine viremia and immune response). On day 29, after vaccination, the viral challenge was given (ZIKV strain PRVABC59 @10^6^ PFU). After 48 h, mice were bled again to determine viremia and then at various time points, mice were sacrificed and necropsied (along with preservation and examination of various organs).	Robust humoral and cell-mediated immune response; protected mice from the virus challenge; also protected testes (in vaccinated males) following challenge.	IM or a combination of IM and electroporation (EP)	[273]
ZIKV LAV (having a nine-residue capsid protein deletion (C7)	Live-attenuated vaccine (LAV)	Nine amino-acid deletions in the viral capsid protein.	Three-week-old A129 mice were vaccinated with a single dose (10^5^ FFU) of the vaccine; 28 days following vaccination, blood was collected (to measure immune response) and then challenged with ZIKV PRVABC59 strain (10^6^ PFU).	Robust humoral immune response; protected mice from the virus challenge; also prevented ZIKV transmission to fetus (from mother).	Subcutaneous (SC)/Intracranial	[274]
ZIKV NS4B protein mutant (s)	Live attenuated vaccine (LAV)	Site-directed mutagenesis (in viral NS4B) was used to create mutants. C100S was observed to be more attenuated than the other 2 mutants (NS4B-C100A and NS4B-P36A). C100S also maintained good immunogenicity.	WT B6 and AB6 mice (5–6-week-old) were inoculated with WT ZIKV FSS13025ic and its mutants (50 to 1 × 10^4^ FFU). Various assays (neutralization, passive immunization, and T-cell depletions), assays were used to determine the vaccine efficacy.	Induction of humoral immunity and cell-mediated immunity CD4+ and CD8+ T cell responses.	Intraperitonial (IP)	[275]
ZIKV carrying mutations in E (N154A) and NS1 (N130A and N207A)	Live attenuated vaccine (LAV)	Mutations were created by employing site-directed mutagenesis.	Three to four week old mice (A129) were vaccinated (1000 PFU of recombinant MR766 [rMR] or the mutant [m2MR and m5MR] viruses diluted in 100 μL of PBS). A total of 28 days following vaccination, mice were challenged with the 10,000 PFU of rMR. Blood was collected for determining immune response.	Robust humoral and cell-mediated immune response; protected mice from the virus challenge.	Subcutaneous (SC)	[276]
Codon pair-deoptimized ZIKVs	Live attenuated vaccine (LAV)	Three codon pair-deoptimized ZIKVs (Min E, Min NS1, and Min E+NS1). The amino acid sequence of these viruses are 100% preserved.	AG6 mice lacking the type I and type II IFN (IFN-α/β/γ) receptors were inoculated with the vaccine strains (@10^4^, 10^3^, 10^2^, 10^1^, or 100 PFU). At 3- and 6-days post vaccination (DPV), viremia was accessed. At various time points, various body tissues were collected for examination and viral load estimation. A total of 28 DPV mice were challenged with ZIKVwt(@10^4^ IFU). A total of 2 days post challenge, mice were bled for viremia determination and 14 days post challenge, all mice were bled (for determining humoral immune response) and sacrificed.	Min E+NS1, caused induction of sterilizing immunity and induction of strong humoral immune response; single vaccine dose was found effective (in response to challenge and preventing ZIKV transmission to fetus from mother).	Intraperitonial (IP)	[277]
Attenuated ZIKV (rGZ02a)	Live attenuated vaccine (LAV)	Live attenuated ZIKV vaccine	6-week-old female C57BL/6 mice were s.c. immunized twice with 1 × 10^4^ (low), 1 × 10^5^ (medium), or 1 × 10^6^ (high) FFU of rGZ02a at a 3-week interval. Humoral immune response was measured 2- and 8.5-weeks post vaccination.	Medium and high doses induced a long lasting (at least 8.5 weeks) and robust humoral immune response; neonates born from vaccinated dams remain free of brain damage and neurological disorders; rGZ02a vaccine also exhibited good anamnestic immune response when primed by Ad2-prME; ZIKV vaccine having prM and E proteins.	Subcutaneous (SC)/Intraperitonial (IP)	[278]
Live-attenuated ZIKV strain (named Z7)	Live attenuated vaccine (LAV)	Modified 5′ UTR (by inserting 50 amino acids) of a pre-epidemic ZIKV Cambodian strain, FSS13025	Four-week-old Ifnar1-/- mice (in C57BL/6J background) were subcutaneously injected with 1 × 10^5^ FFU of Z1 or Z7 (G10) in phosphate buffer saline (PBS) on D0. Serum samples were collected on day 24. On D42 p.i., the mice were challenged with 1 × 10^5^ PFU of ZIKV (strain PRVABC59), and blood was collected from D1 to D3 post-challenge (to measure the viremia). On D3 p.c., the mice were euthanized, and the liver and spleen were collected to measure the viral load.	Robust humoral and cellular immune responses that prevented viremia after the challenge (with a high dose of an American epidemic ZIKV strain PRVABC59 infection)	Subcutaneous (SC)	[279]
**DNA Vaccines**						
**Zika Virus** **Strain**	**Vaccine Type**	**Development Strategy**	**Study Conducted**	**Mechanism**	**Route**	**Reference**
ZIKV Puerto Rico Strain (PRVABC59)	DNA Vaccine	DNA vaccine containing prM and E protein.	Vaccination was done in Ifnar1-/- mice (2 doses, 2 weeks apart). Following the challenge (Puerto Rico Strain PRVABC59), the mice did not demonstrate the testicular damage and sperm deterioration, proving it a safe vaccine candidate.	Humoral and T Cell immunity	Intramuscular (IM)	[280]
ZIKV Puerto Rico Strain PRVABC59, West African IbH 30656 and East African MR766 (challenge strains)	DNA Vaccine	Amphiphilic block copolymer (ABC) encoding the sequence of prM and E.	Vaccination was carried out in C57BL/6c mice @ Day. 0, 24, 42, and 199. With the challenge of various ZIKV isolates (Puerto Rico Strain PRVABC59, West African IbH 30656, and East African MR766),	Humoral and T Cell immunity in a dose-dependent manner (low protection was observed at 5 ug but high protection from diseases was observed at 10 and 50 ug).	Intramuscular (IM)	[281]
ZIKV SMGC-1 strain (Challenge)	DNA Vaccine	DNA vaccine encoding pVAX1-ZME of ZIKV.	Three doses of vaccine (50 ug of pVAX1-ZME) were inoculated (3 weeks apart) in immunocompetent BALB/c mice, and it was shown that mice exhibited potent humoral and CMI and also prevented the transfer of infection from mother to offspring.	Induction of both Th1 and Th2 responses and cytokines (IL-2, IL-4, IL-5, and IFN-γ).	In vivo electroporation	[282]
**ZIKV Subunit Vaccines**						
**Zika Virus** **Strain**	**Vaccine Type**	**Development Strategy**	**Study Conducted**	**Mechanism**	**Route**	**Reference**
ZIKV Puerto Rico Strain PRVABC59	Subunit Vaccine	Subunit ZIKV vaccine containing E Protein.	Vaccine was inoculated in 3 different mice models; BALB/c, C57BL/6, and Swiss Webster mice (3 doses at 3 week intervals). The vaccine caused the induction of higher levels of the antibodies that protected the mice from developing clinical infection in challenge with Puerto Rico Strain PRVABC59. CoVaccine HT-based vaccine induced higher antibody responses as compared to the alum adjuvanted vaccines.	Robust Humoral Immune response.	Intramuscular (IM)	[283]
ZIKV Puerto Rico Strain PRVABC59	Subunit Vaccine	Subunit ZIKV vaccine containing E Protein.	Vaccine was inoculated in non-human primate (*Cynomolgus macaques*) with 3 doses at 3 week intervals and demonstrated the strong humoral response following vaccination and also prevented the maternal infection transfer to the fetus.	Robust Humoral Immune response.	Intramuscular (IM)	[284]
ZIKV Puerto Rico Strain PRVABC59	Subunit Vaccine	Subunit ZIKV vaccine containing ED-III fragments.	ED III domain was produced in *E. coli*. A total of 4 doses of vaccines were inoculated in C57BL/6 mice and resulted in higher levels of IL-4, IL-6, and IFN-γ. Vaccine also elicited good humoral immune response in mice.	Robust Humoral Immune response.	Intra-Peritoneal (IP)	[285]
ZIKV 2015/Honduras (R103451) and 2015/Colombia (FLR) Challenge	Subunit Vaccine	Subunit ZIKV vaccine containing ED-III different fragments.	To compare the immunogenicity of 3 different ED-III, residues 296–406, 298–409, and 301–404 of the ZIKV envelope (E) protein domain III (EDIII) fused with a C-terminal Fc of human IgG and inoculated in BALB/c mice and A129 mice (Five doses at days 0, 21, 42, 210, and 300). The vaccine having ED-III residues 298–409 elicited most strong humoral immune response. The immunity was transferred from mother to offspring and also protected the pups from ZIKV infection on challenge.	Robust Humoral Immune response.	Intramuscular (IM)	[286]
**Viral Vector Based ZIKV Vaccines**						
ZIKV Puerto Rico strain PRVABC59	Viral Vector	ZIKV prM-E gene expression in Ad4 and Ad5 (to form Ad4-prM-E and Ad5-prM-E, respectively).	Vaccination was done in immunodeficient mice and C57BL/6 mice (2 doses at 3 week intervals). Ad4-prM-E induced only CMI whereas Ad5-prM-E induced both humoral and CMI. On challenge with virulent ZIKV in C57BL/c, all mice survived indicating the protective role of both Ad4-prM-E and Ad5-prM-E in inducing protective immunity levels.	Induced both humoral and CMI.	Intramuscular (IM)	[287]
Brazilian ZIKVand Puerto Rico strain PRVABC59	Viral Vector	RhAd52-prMEnv.	ZIKV vaccine inoculation was done in rhesus macaques and elicited good humoral immunity in rhesus macaques.	Humoral immunity	Intramuscular (IM)	[288]
ZIKV Cambodian strain FSS13025	Viral Vector	(rVSV)-based ZIKV prM-E-NS1 vaccine.	Single dose of vaccine in A129 and BALB/c mice induced protective humoral and CMI that protected the mice partially on ZIKV challenge.	Humoral and CMI	Intramuscular (IM)	[289]
ZIKV Puerto Rico strain PRVABC59	Viral Vector	VSV-Capsid and VSV-ZikaE260–425.	Single dose of both vaccines was done in BALB/c mice and both vaccines induced good humoral immune response however, however, vaccine containing capsid region induced stronger T cell immunity as compared to the vaccine containing E260–425.	Humoral and CMI	Intramuscular (IM)	[290]

“IM” indicates Intra-Muscular, “IP” indicates Intraperitoneal, “EP” indicates Electroporation, and “SC” indicates Subcutaneous routes of vaccine administration/inoculation.

**Table 4:** Strategies to control mosquitoes/vectors involved in ZIKV transmission.

**Sr No. 1**	**Chemical Control**				
	**Compound/Organism**	**Vectors**	**Mechanism**	**Remarks**	**Reference**
	Pyrethroids	*Aedes aegypti*	Disruption of voltage-gated sodium channels in neuronal membranes, thereby disrupting the electrical signaling in the insect nervous system.	Emergence of resistance	[319]
	Organophosphorus	*Aedes aegypti*	Inhibition of acetylcholine esterase in insects.	Emergence of resistance	[320]
	Insect growth regulators	*Aedes albopictus*	Inhibiting specific biochemical pathways essential for insect growth and development.	Emergence of resistance	[321]
	Carbamates (propoxur, bendiocarb)	*Aedes aegypti*, *Aedes albopictus*	Inhibition of acetylcholine esterase in insects.	Emergence of resistance	[322,323]
**Sr No. 2**	**Herbal Control**				
	**Compound/Organism**	**Vectors**	**Mechanism**	**Remarks**	**Reference**
	*Illicium verum* (EO)	*Aedes aegypti*, *Aedes albopictus*	Morphological aberrations at death in larvae and pupae.	--	[302]
	*Zanthoxylum limonella* (EO)	*Aedes aegypti*, *Aedes albopictus*	Morphological aberrations at death in larvae and pupae.	--	[302]
	*Cymbopogon citratus*, *Cymbopogon winterianus*	*Aedes aegypti*	Good larvicidal activity.	--	[303]
	*Eucalyptus citriodora*, *Eucalyptus camaldulensis*	*Aedes aegypti*	Good larvicidal activity.	--	[303]
	*Achillea biebersteinii*, *Juniperus procera*	*Aedes aegypti*	Adult stage was more sensitive than larvae.	--	[304]
	*Annona glabra* (nanoparticles)	*Aedes aegypti*, *Aedes albopictus*	*Aedes albopictus* found to be more susceptible	--	[305]
	*Pavetta tomentosa*, *Tarenna asiatica.*	*Aedes aegypti*	Larvicidal and adulticidal activity.	--	[306]
	*Ambrosia arborescens* (nanoparticles)	*Aedes aegypti*	Good larvicidal activity.	--	[307]
	*Lippia alba*, *L. origanoides*, *Eucalyptus citriodora*, *Cymbopogon flexuosus*, *Citrus sinensis*, *Cananga odorata*, *Swinglea glutinosa*, *Tagetes lucida*	*Aedes aegypti*	Pupcidal and adulticidal activity.	--	[308]
**Sr No. 3**	**Control through Entomopathogenic Bacteria**				
	**Compound/Organism**	**Vectors**	**Mechanism**	**Remarks**	**Reference**
	*Bacillus thuringiensis israelensis* (*Bti*)	*Aedes aegypti*, *Aedes albopictus*	Good larvicidal activity.	Bacteria-derived toxin kills mosquitoes.	[310,324,325,326,327,328]
	*Wolbachia*	*Aedes aegypti*	-Reducing cellular resources for viral replication -immune-priming -induction of phenoloxidase -miRNA-dependent immune pathway.	Less chances of resistance development.	[311,329,330,331]
**Sr No. 4**	**Control through Entomopathogenic Fungi**				
	**Compound/Organism**	**Vectors**	**Mechanism**	**Remarks**	**Reference**
	*Metarhizium anisopliae*	*Aedes aegypti*, *Aedes albopictus*	Fungal hyphae penetration leads to mosquito death.	Cost effective and less chances of resistance development.	[312,332,333]
	*Beauveria bassiana*	*Aedes aegypti*, *Aedes albopictus*	Fungal toxins (beauvericin A or F, beauvericin E) kill the mosquito.	--	[313,334,335,336]
	*Aspergillus flavus*, *Aspergillus fumigatus*, and *Aspergillus terreus*	*Aedes aegypti*,	--	--	[314,315,337]
**Sr No. 5**	**Control through *Toxorhynchites* Mosquitoes**				
	**Compound/Organism**	**Vectors**	**Mechanism**	**Remarks**	**Reference**
	*Toxorhynchites splendens*, *Tx. amboinensis*, and *Tx. moctezuma*	*Aedes aegypti*, *Aedes albopictus*	Larvae voraciously eat the larvae of *Aedes aegypti* and *Aedes albopictus.*	Share the same environment and is therefore used effectively for biological control of *Aedes* spp. mosquitoes.	[316,338,339,340]
**Sr No. 6**	**Control through Fishes**				
	**Compound/Organism**	**Vectors**	**Mechanism**	**Remarks**	**Reference**
	*Poecilia reticulata*, *Rasbora daniconius*, *Aplocheilus dayi*, *Oriochromis mossambicus*, *O. niloticus*, and *Puntius bimaculatus*	*Aedes aegypti*	Engulf mosquito larvae and pupa.	Share the same environment and are therefore used effectively for the biological control of mosquitoes.	[341]
	Chinese cat fish	*Aedes aegypti*			[342]
	*Poecilia reticulata*, *Puntius bimaculatus*, and *Rasbora caveri*	*Ae. albopictus*			[343]
	*Gambusia affinis*	*Aedes* sp.			[344]
	*Melanotaenia splendida splendida*	*Aedes aegypti*			[345]
**Sr No. 7**	**Control through Tadpoles**				
	**Compound/Organism**	**Vectors**	**Mechanism**	**Remarks**	**Reference**
	*Bufo*, *Ramanella*, *Euphlyctis, and Hoplobatrachus* (genera used)	*Aedes aegypti*	Various tadpoles (belonging to various genera) effectively engulfed the mosquito eggs.	Share the same environment and are therefore used effectively for the biological control of mosquitoes.	[346]
	*Hoplobatrachus tigerinus*	*Aedes aegypti*	Effective engulfment of mosquito larvae.		[347]
**Sr No. 8**	**Control through Copepods**				
	**Compound/Organism**	**Vectors**	**Mechanism**	**Remarks**	**Reference**
	*Mesocyclops aspericornis*,	*Aedes aegypti*	Effective engulfment of mosquito larvae.	Share the same environment and therefore is used effectively for the biological control of mosquitoes.	[348]
	*Macrocyclops albidus*	*Ae. albopictus*			[349]
	*Mesocyclops thermocyclopoide*	*Aedes aegypti*			[350,351]
**Sr No. 9**	**Control through Genetic Tailoring/Modification**				
	**Compound/Organism**	**Vectors**	**Mechanism**	**Remarks**	**Reference**
	*Ae. aegypti* OX513A	*Aedes aegypti*	Compete natural mosquitoes for food and reproduction and release of males leads to >95% decline in larval and pupal populations.	Cost and labor-intensive.	[317,318,352,353,354,355]
	*Ae. aegypti* OX5034	*Aedes aegypti*		Cost and labor-intensive.	[356]
**Sr No.10**	**Control through Sterile Insect Techniques (SIT)**				
	**Compound/Organism**	**Vectors**	**Mechanism**	**Remarks**	**Reference**
	Release of sterile males in the mosquito habitat.	*Aedes aegypti*, *Aedes albopictus*	Disruption of reproductive cycle; sterile males inseminate wild females, and no progeny is produced.	The technique is specific and ecological friendly.	[357,358,359,360,361,362,363]

**Table 5:** Synthetic antivirals tested against Zika Virus.

Direct Acting Antivirals				
Sr No.	Compound Name	Viral Target Inhibition	Results	Reference
1	Sofosbuvir	Pan-methyltransferase	Demonstrated in vitro antiviral activity	[364] [365] [366]
2	NITD008	Pyrimidine synthesis	Demonstrated in vitro and in vivo antiviral activity	[367] [368]
3	Suramin	NS3	Demonstrated in vitro antiviral activity	[369]
4	Temoporfin and novobiocin	NS2B-NS3 protease	Demonstrated in vitro and in vivo antiviral activity	[370] [371]
5	Myricetin, luteolin, isorhamnetin, apigenin, curcumin, niclosamide, and nitazoxanide.	NS2B-NS3 protease	Demonstrated in vitro antiviral activity	[372] [370] [371]
6	7-deaza-2-CMA, Sofosbuvir, and BCX4430	RNA dependent RNA polymerase	Demonstrated in vitro and in vivo antiviral activity	[373] [374] [375]
	**Antivirals inhibiting the host targets**			
	**Compound name**	**Host Target Inhibition**	**Results**	**Reference**
1	Ribavirin	Purine synthesis	Demonstrated in vitro and in vivo antiviral activity	[376]
2	Azathioprine	Purine synthesis	Demonstrated in vitro antiviral activity	[217]
3	6-azauridine, 5-fluorouracil	Pirimidine synthesis	Demonstrated in vitro antiviral activity	[377]
4	Chloroquine	pH-dependent steps of viral replication	Demonstrated in vitro antiviral activity	[378]
5	Saliphenylhalamide (SaliPhe)	Viral entry	Demonstrated in vitro antiviral activity	[379]
6	Memantine, MK-801, agmatine, and ifenprodil	Neuronal cell death	Demonstrated in vitro and in vivo antiviral activity	[380]

**Table 6:** Medicinal/natural plants possessing anti-ZIKV activities.

Sr No.	Compound Name	Source	Mode of Action	Results	Reference
1	Labyrinthopeptin	*Actinomadura namibiensis*	Viral envelope disruption	Demonstrated in vitro antiviral activity	[382]
2	Epigallocatechin	Green Tea	Inhibits viral entry, inhibition of NS3, and NS2B3 protease	Demonstrated in silico antiviral activity	[383] [384] [385,386] [387] [388]
3	Gossypol	Plants of the *Gossypium* genus	--	--	[389] [390]
4	Resveratrol	Various plant species	Inhibits viral replication	Demonstrated in vitro antiviral activity	[391] [392]
5	Berberine	*Berberis vulgaris*	--	Demonstrated in vitro antiviral activity	[393]
6	Cephalotaxine	*Cephalotaxus drupacea*	Inhibits viral replication	Demonstrated in vitro antiviral activity	[394]
7	Nanchangmycin	*Streptomyces nanchangensis*	Inhibits clathrin-mediated endocytosis	Demonstrated in vitro antiviral activity	[395]
8	Quercetin	Various plant species	--	Demonstrated in vitro antiviral activity	[396]
9	Sophoraflavenone G	*Sophora flavescens*	RNA polymerase interference	in vitro antiviral activity	[397]
10	Pinocembrin	Honey and tea	Inhibits the synthesis of viral E and RNA	in vitro antiviral activity	[398]
11	Sinefungin	*Streptomyces griseoleus*	Inhibits viral RNA cap methylation	Demonstrated in silico and in vitro antiviral activity	[399]
12	Cavinafungin	*Colispora cavincola*	Inhibits host signal peptidase (in ER), thus inhibiting viral synthesis	in vitro antiviral activity	[400]
13	Emetine	*Carapichea ipecacuanha*	Inhibition of viral NS5 RdRp activity	in vitro antiviral activity	[401]
14	Lycorine	*Amaryllidaceae* species	Inhibition of viral NS5 RdRp activity	Demonstrated in vitro and in vivo antiviral activity	[402]
15	Naringenin	Citrus fruits	Inhibition of viral replication and assembly	in vitro antiviral activity	[403]
16	Delphinidin	Various fruits	Inhibition of viral attachment and entry in host cells	in vitro antiviral activity	[388]
17	Baicalein	*Scutellaria baicalensis* & *Scutellaria lateriflora*	--	Demonstrated in silico antiviral activity	[404]
18	Baicalin	*Scutellaria baicalensis* & *Scutellaria lateriflora*	--	Demonstrated in silico antiviral activity	[404]
19	Ginkgolic acid	*Ginkgo biloba*	Inhibits the viral fusion	In vitro antiviral activity	[405]
